# Experimental Infection of Pigs with a ST 245 *Brachyspira hyodysenteriae* Isolated from an Asymptomatic Pig in a Herd with No History of Swine Dysentery

**DOI:** 10.3390/vetsci9060286

**Published:** 2022-06-10

**Authors:** José Paulo H. Sato, Amanda G. S. Daniel, Carlos E. R. Pereira, Mariana R. Andrade, Ricardo P. Laub, Michelle P. Gabardo, Luisa V. A. Otoni, Nubia R. Macedo, Javier A. Barrera-Zarate, Roberto M. C. Guedes

**Affiliations:** Department of Veterinary Clinic and Surgery, Veterinary School, Universidade Federal de Minas Gerais, Belo Horizonte 130161-970, Brazil; zpsato@hotmail.com (J.P.H.S.); amandavet2007-1@hotmail.com (A.G.S.D.); carlos.pereira@ufv.br (C.E.R.P.); marianandrade@hotmail.com (M.R.A.); ricardolaub@gmail.com (R.P.L.); michelle.gabardo@ifmg.edu.br (M.P.G.); luisavianna.vet@gmail.com (L.V.A.O.); nubia@iastate.edu (N.R.M.); jabarreraz@unal.edu.co (J.A.B.-Z.)

**Keywords:** swine dysentery, subclinical *Brachyspira hyodysenteriae*, experimental infection, diarrhea, pig

## Abstract

Swine dysentery (SD) is characterized by a severe mucohemorrhagic colitis caused by infection with *Brachyspira* species. In infected herds the disease causes considerable financial loss due to mortality, slow growth rates, poor feed conversion, and costs of treatment. *B. hyodysenteriae* is the most common etiological agent of SD and infection is usually associated with disease. However, isolated reports have described low pathogenic strains of *B. hyodysenteriae*. The aim of this study was to describe an experimental infection trial using a subclinical *B. hyodysenteriae* isolated from an animal without clinical signs and from a disease-free herd, to evaluate the pathogenicity and clinical pathological characteristics compared to a highly clinical isolate. Forty-eight 5-week-old pigs were divided into three groups: control, clinical and the subclinical isolates. The first detection/isolation of *B. hyodysenteriae* in samples of the animals challenged with a known clinical *B. hyodysenteriae* strain (clinical group) occurred 5th day post inoculation. Considering the whole period of the study, 11/16 animals from this group were qPCR positive in fecal samples, and diarrhea was observed in 10/16 pigs. In the subclinical isolate group, one animal had diarrhea. There were SD large intestine lesions in 3 animals at necropsy and positive *B. hyodysenteriae* isolation in 7/15 samples of the subclinical group. In the control group, no diarrhea, gross/microscopic lesions, or qPCR positivity were observed. Clinical signs, bacterial isolation, macroscopic and histologic lesions were significantly difference among groups, demonstrating low pathogenicity of the subclinical isolate in susceptible pigs.

## 1. Introduction

*Brachyspira hyodysenteriae* is the etiological most common agent of swine dysentery (SD), characterized by mucohemorrhagic colitis [1,2]. Clinical signs range from moderate mucoid to bloody diarrhea, with a mortality rate ranging from 30 to 90%. SD gross lesions include multifocal mucosal necrosis and hemorrhage, excess of mucus, associated with fibrinous exudate and thickening of the mucosa of variable intensity. Microscopically, changes in the cecum and colon characterized by goblet cell hyperplasia, hemorrhage, superficial necrosis, and neutrophilic inflammatory infiltrate in the lamina propria [2].

In recent years there has been reported a reemergence of the disease in several countries and the emergence of two new species, *B. hampsonii* and *B. suanatina*, with similar pathogenic characteristics of SD [3,4,5,6]. Atypical isolates of *B. hyodysenteriae* were previously described and characterized as low pathogenic, capable of colonizing but not inducing clinical disease [7,8,9]. Studies evaluating pathogenic and molecular characteristics of atypical strains isolated from *B. hyodysenteriae* are scarce from herds without clinical disease [10,11].

This study aimed to describe an experimental infection trial in pigs using a subclinical *B. hyodysenteriae* isolate obtained from an animal without clinical signs and from an apparently healthy herd with no history of SD. The pathogenicity, clinical pathological and molecular findings of this subclinical isolate of *B. hyodysenteriae* compared to a highly clinical isolate were evaluated.

## 2. Materials and Methods

### 2.1. Animals and Experimental Design 

This study was approved by the Ethics Committee on Animal Experimentation of the Universidade Federal de Minas Gerais (Approval Number: CEUA #177/2015).

Forty-eight 5-week-old piglets (7.95 ± 1.29 kg/wt) were obtained from a commercial farm with no history of disease associated with *Brachyspira* spp., *Lawsonia intracellularis* or *Salmonella* sp. The animals were randomly divided into three groups (16 animals/group): control (CTRL), clinical isolate (CLIN) and subclinical isolate (SUBCL), which were kept in 4 pens (4 animals/pen) in three separated rooms throughout the experiment. 

During a 7-days acclimatation period, the pigs were tested for *Brachyspira* spp. and *Salmonella* sp. by culture of fecal samples and *L. intracellularis* by PCR testing. Room temperature and humidity among room were the same. Feed and water were available *ad libitum* for all treatments. Parenteral or oral medications were not used throughout the study period. The daily management activities in each group were performed using strict biosecurity protocols, including unique instruments and different personal to avoid cross-contamination.

### 2.2. Inoculum 

Both isolates used in this study were obtained from two different Brazilian pig farms in 2013 and were frozen at −80 °C in the Laboratory of Molecular Pathology at the Veterinary School of the Universidade Federal de Minas Gerais (UFMG). The clinical isolate used in the CLIN group was obtained from a clinically affected pig with mucohemorrhagic diarrhea and colitis from a SD positive herd. The subclinical isolate was obtained from an animal from a herd with no clinical signs or history of SD. No antimicrobials were being used in this herd, so there was no possibility to mask clinical signs of the disease. Phenotypically, both isolates produced strong hemolysis in blood agar. Both isolates were identified as *B. hyodysenteriae* based on PCR [12] and *nox* gene sequencing [5]. The multilocus sequence typing (MLST) analysis of these isolates classified both as sequence type (ST) 245 [13].

Isolates were cultured on trypticase soy agar with 5% sheep blood (TSA) containing 12.5 mg/L of rifampicin, 200 mg/L of espectinomicin, 50 mg/L of vancomicin and 12.5 mg/L of colistin [14], under anaerobic conditions with N2 (80%), CO2 (10%) and H2 (10%), at 42 °C and examined for growth at 72 and 96 h. 

After growth, the agar plates were washed with PBS and the supernatant was incubated in trypticase soy broth (TSB), enriched with 0.5% glucose, 0.2% NaHCO3, 0.05% L-cysteine-HCl, 1.0% yeast extract, 10% fetal bovine serum and 5% swine fecal extract [15] in a ratio of 1:100 mL (wash:broth) for 24 h at 42 °C in a shaker, followed by inoculation of the animals.

### 2.3. Animal Inoculation 

All pigs were inoculated by intragastric gavage for three consecutive days as previously described [16,17]. Pigs in the CLIN and SUBCL groups received 50 mL of the inoculum at concentrations determined by qPCR of 5.1 × 10^7^, 2.1 × 10^8^ and 2.2 × 10^8^ bacteria/mL (CLIN group) and 1.2 × 10^8^, 2.3 × 10^8^ and 1.3 × 10^8^ bacteria/mL (SUBCL group) in three consecutive days, respectively. The CTRL group was inoculated with 50 mL of sterile TSB. To decrease gastric transit time, feed was removed for 16 h prior to, and returned one hour after inoculation [17]. 

### 2.4. Clinical Evaluation and Sample Collection

After inoculation, all animals were observed twice a day for evaluation of clinical signs of diarrhea, and fecal consistency based on the following score: 0 = normal, 1 = semi-solid consistency, 2 = creamy consistency and 3 = watery consistency, with addition of 0.5 for the presence of detectable mucus and/or blood.

The quantitative evaluation of *B. hyodysenteriae* elimination in the feces were performed by qPCR on samples collected at −7, 5, 7, 11, 15 and 18 days post inoculation (DPI). *Brachyspira* spp. isolation in selective medium as described previously were analyzed in fecal samples collected at −7, 0, 3, 5, 7, 9, 11, 13, 15 and 18 DPI. 

### 2.5. DNA Extraction and qPCR

DNA from fecal samples were extracted using a commercial kit (QIAamp DNA Stool kit−Qiagen Inc., Toronto, ON, Canada) according to the manufacturer’s instructions. The amount of *B. hyodysenteriae* DNA was determined by real-time PCR using published primers [17]. One gram of negative feces and the addition a known number of *B. hyodysenteriae* (10^1^–10^8^ bacteria/gram of feces) was used to determine the standard curve. Threshold values were considered for detection of *Brachyspira* determined by 10^3^–10^8^ bacteria/gram of feces, using the regression equation from the standard curve (R^2^ = 0.990).

The reaction was performed in a final volume of 25 μL, consisting of 1x SYBR Green PCR Master Mix, 1x QN ROX Reference Dye (Quantiia SYBR Green PCR Kit, Qiagen Inc., Toronto, ON, Canada), 500 nM of each primer and 5 μL of DNA. The samples were placed in 96-well plates and amplified in the ViiATM 7 Real-Time PCR System (Applied biosystems) thermocycler, with amplification conditions: 2 min at 50 °C, 10 min at 95 °C, 40 cycles of 15 sec at 95 °C and 1 min at 60 °C. All reactions were performed in duplicate, and each reaction included the standard curve and negative control, being analyzed in QuantStudio TM Real-Time PCR v1.2 software (Thermo Fisher Scientific, Waltham, MA, USA).

PCR specificity was tested against *Bacteroides fragilis*, *B. murdochii*, *B. pilosicoli*, *Clostridioides difficile*, *C. perfringens*, *Enterococcus faecalis*, *Escherichia coli*, *L. intracellularis*, *Pseudomonas aeruginosa* and *Salmonella* sp.

### 2.6. Necropsy

Euthanasia and necropsy were performed when the animals were clinically debilitated according to the criteria of the CEUA or at the end of the study, on 18 DPI. For macroscopic and microscopic evaluation, segments of the small intestine, large intestine (cecum, proximal and spiral colon) and mesenteric lymph nodes were analyzed and fixed in 10% buffered formalin.

### 2.7. Macroscopic Evaluation

Macroscopically, cecum and colon were evaluated for the presence of edema, excessive mucus in the lumen, mucosal hemorrhage and fibrinous exudate.

### 2.8. Histology

All sampled fragments were processed according to routine histological techniques and stained with hematoxylin and eosin (H&E) [18]. The presence of superficial necrosis, hemorrhage, goblet cell hyperplasia, crypt abscesses and neutrophil infiltrate in the lamina propria were evaluated in the cecum and colon, with lesions scored as zero (no lesion) to three (severe diffuse lesion). The final score was determined by the sum of the five parameters evaluated and classified as mild (<5), moderate (5 and <10) and severe (≥10), with a maximum value of 15. All histological sections were evaluated by two pathologists blinded about experimental groups, and the mean of these two evaluations was used in the analyzes.

### 2.9. Fluorescence In Situ Hybridization (FISH) 

Sections of the large intestines were used for FISH, according to Jensen et al. [19] with probes specific to *B. hyodysenteriae* [20]. Presence of *B. hyodysenteriae* was classified as mild (+), moderate (++) or intense (+++), according to the ratio of labeled spirochetes.

### 2.10. Bacterial Isolation

At the end of the study period, fecal samples, small intestinal contents and mucosal scrapings of the cecum and colon were collected for bacterial isolation. For *Brachyspira* spp., feces and scrap samples from the large intestine were culture as described above. Samples of the small intestine were seeded on blood and MacConkey agar for evaluation of enterotoxigenic *E. coli* and Rappaport broth and Hectoein agar for *Salmonella* sp. 

### 2.11. Statistical Analysis

The SPSS software v19.0 (SPSS Inc., Chicago, IL, USA) was utilized to perform all analyses. The presence or absence of mucohemorrhagic diarrhea, macroscopic lesions and histopathological lesion scores among groups were compared using Kruskal–Wallis test, with *p*-values < 0.05 considered significant.

## 3. Results

### 3.1. Clinical Evaluation

During the acclimation period, one animal from the SUBCL group suddenly died and was excluded from the study. At the necropsy, valvular endocarditis was diagnosed. All other animals had normal or semi-solid fecal consistency (score 0 or 1) and all fecal samples were negative in bacterial isolation for *Brachyspira* spp. or *Salmonella* sp. and were negative by PCR for *L. intracellularis*. 

Aqueous and/or mucohemorrhagic diarrhea (score ≥ 3) was first observed in the 7th DPI in tree animals (#2, #11 and #12) of the CLIN group. Considering all study period 10/16 animals in this group had diarrhea. In the SUBCL group, only one animal (#25) had mucohemorrhagic diarrhea starting on the 15th DPI. None of the animals in the CTRL group had any clinical signs of diarrhea during the study period (Table 1). Considering days with diarrhea, an animal from the CLIN group had diarrhea for 11 days and in the SUBCL group, a single pig had diarrhea for three days. Three clinically debilitated animals (#2, #6 and #13) from CLIN group were euthanized at 13, 13 and 16 DPI, respectively. Significant differences of clinical signs of diarrhea were observed between the CTRL and the other two groups (SUBCL and CTRL) (Table 2).

### 3.2. Anatomopathological Analysis

#### 3.2.1. Gross Lesions

**Figure 1:**
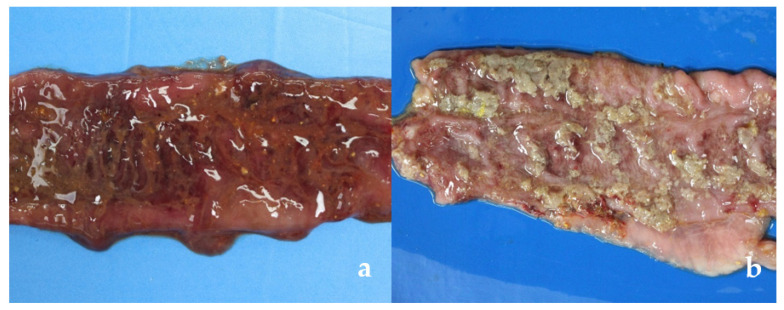

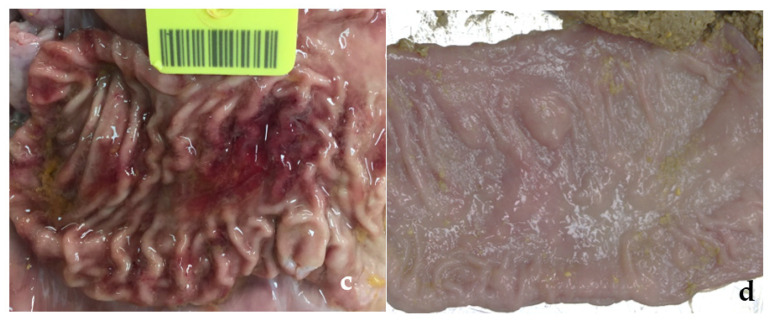
Gross lesions in the spiral colon. (**a**,**b**) Clinical isolate (CLIN group), excessive luminal mucus with diffuse mucosa hemorrhage (**a**), and superficial fibrinonecrotic exudate (**b**). (**c**) Subclinical isolate (SUBCL group), discrete focal increase in luminal mucus and moderate multifocal mucosal hemorrhage. (**d**) Negative control (CTRL group), no gross lesions.

Gross pathological findings were compatible with observed clinical signs. Lesions were more frequent in segments of the spiral colon in pigs with aqueous and/or mucohemorrhagic diarrhea (fecal score ≥ 3) from CLIN group (Table 1). Significant differences were observed between the CLIN and the other two groups (SUBCL and CTRL) (Table 2), more severe in the CLIN animals. Nine and four animals of the CLIN and SUBCL groups, respectively, had macroscopic alterations (Table 1) characterized by luminal mucus, mucosal hemorrhage, necrosis and/or fibrous exudate (Figure 1).

One animal of the CLIN group (#16: edema, hemorrhage, thickening and diffuse marked necrosis of the mucosa) and two of the SUBCL group (#24: edema, hemorrhage and thickening of moderate multifocal mucosa and #26: edema and mucosal hyperemia multifocal) had macroscopic lesions, but no clinical signs were observed. 

#### 3.2.2. Histopathology and FISH

Histological findings are demonstrated in Table 1. In the CLIN group, all animals had lesions based on the evaluated parameters (superficial necrosis, hemorrhage, goblet cell hyperplasia, crypt abscesses and neutrophils infiltrate in the lamina propria). In this group the lesions were more severe and extensive, 8/16 animals were classified with a score >10 with severe lesions. Animal #25, was the only one with high score (13.5) in the SUBCL group and the only one that showed clinical signs of mucohemorrhagic diarrhea that started at 15 DPI. In the CRTL group, two animals had mild infiltration of inflammatory cell in the large intestine, with a score of 1, and no *Brachyspira* spp. associated. No significant lesions were observed in the small intestine and mesenteric lymph nodes in any of the experimental animals. Animals from group CLIN had more histological lesions than the other two groups, and pigs from SUBCL had more lesions than the CTRL group (*p* < 0.05) (Table 2).

**Figure 2:**
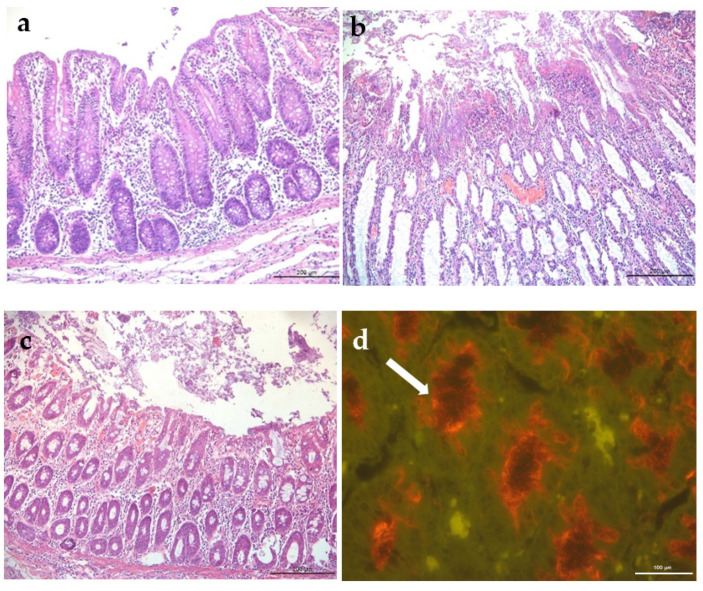
Histologic findings in the colon of pigs from the control or inoculated with *Brachyspira hyodysenteriae* groups. (**a**) Negative control group (CTRL), no visible lesions (H&E). (**b**) Clinical group (CLIN), hemorrhage, diffuse severe superficial necrosis and epithelial detachment, severe inflammatory infiltrate in the lamina propria, H&E. (**c**) Subclinical group (SUBCL), hyperemia, multifocal superficial necrosis with epithelial detachment and moderate inflammatory infiltrate in the lamina propria, H&E. (**d**) Subclinical isolate, spirochetes (arrow) at the apical border and inside enterocytes, fluorescent hybridization in situ (FISH).

FISH assays using probes specific for *B. hyodysenteriae* were positive in 9 animals of CLIN group, all of these with histologic score ≥9.5 in H&E evaluation. Three animals (#24, #25 and #26) from SUBCL group were positive by FISH with 5, 13.5 and 6.5 histologic scores, respectively (Table 1). All animals from CTRL group were negative. Figure 2 shows histological sections of the large intestine of pigs from the three evaluated groups. 

### 3.3. qPCR

The first detection of *B. hyodysenteriae* fecal shedding by qPCR was in animal #2 from CLIN group on the 5th DPI. In this group, considering all study period and the last sample collection (18 DPI), 11/16 animals were positive by qPCR. 

In the SUBCL group, four animals (#20, #24, #25 and #26) were positive starting at 15 DPI. Animal #20 was negative for all evaluated parameters (clinical signs, gross/microscopic lesions and FISH), but positive for qPCR and bacterial isolation of *Brachyspira* spp. Animals #24 and #26 had SD mild macroscopic lesions and moderate histology alterations. Only animal #25 was positive for all parameters evaluated, including mucohemorrhagic diarrhea. 

The qPCR values ranged from 8.5 × 10^3^ to 5.9 × 10^8^ and 2.2 × 10^2^ to 2.5 × 10^7^ organisms per gram of feces in the CLIN and SUBCL groups, respectively. The CRTL group were qPCR negative to *B. hyodysenteriae* in all tested samples.

### 3.4. Bacterial Isolation

Isolation was the most sensitive technique among all parameters used during this study (Table 1). In the CLIN group, *B. hyodysenteriae* isolation from feces of animal #2 was the earliest and coincided with qPCR detection. In this group, 14/16 animals had positive *B. hyodysenteriae* isolation. In the SUBCL group, the first bacterial isolation was obtained from fecal samples of animal #24 at 11 DPI and a total of 7/15 animals had positive isolation on 18 DPI. No growth of *B. hyodysenteriae* was obtained in the CRTL group.

No other clinical bacteria, particularly enterotoxigenic *E. coli* and *Salmonella* sp. were detected in the selective culture media used in samples collected at the time of necropsy.

## 4. Discussion

Low clinical *B. hyodysenteriae* imposes a critical epidemiological risk for contamination of negative herds, as healthy replacement animals originated from well managed hog farms with high healthy sanitary status, might carry the infection to not so well managed herds with other sanitary problems, and SD might manifest. So, the understanding of the magnitude and capacity of low clinical and subclinical *B. hyodysenteriae* isolates to cause SD is imperative. There are some studies describing the detection and isolation of *B. hyodysenteriae* strains from healthy animals from SD free herds [7,8,9,11,21]. However, clinical-pathological characterization of these subclinical isolates in experimental challenge studies is scarce [7].

In the present study, clinical course of mucohemorrhagic diarrhea started at 7 DPI in animals of the CLIN group, with a morbidity of 62.5% in 18 days of evaluation. These findings were similar with Wilcock et al. (1979) that described clinical signs observed at 7–10 DPI. Three animals of the CLIN group were euthanized due to the debilitating conditions caused of SD, demonstrating high pathogenicity of this isolate when compared to the SUBCL isolate. Lysons et al. [7] using different group of pigs challenged with three subclinical strains of *B. hyodysenteriae* did not observe clinical signs of SD in two strains used, even with colonization confirmed by qPCR. In the present study, only one animal of the SUBCL group developed mucohemorrhagic diarrhea at 15 DPI, demonstrating the delayed onset of clinical disease and potential reduced pathogenicity. This difference between the manifestation of subclinical *B. hyodysenteriae* isolates demonstrated the relevance of increasing the knowledge about them and the very likely differences among them. 

Necropsy SD findings characterized by mucoid colitis or mucohemorrhagic and fibrous mucus typhlitis observed in the present study are consistent with the literature [17]. The sum of the histology lesion scores in large intestine classified as moderate to severe (≥5) were in accordance with the presence of gross lesions and the proportion of spirochetes labelled by FISH. La et al. [11] reported *B. hyodysenteriae* recovered from pigs without clinical signs and, on intestinal histological evaluation of three animals, no lesions suggestive of SD were observed. In the present study, spirochetes upon histology evaluation were observed in samples with more pronounced gross and microscopic lesions.

Isolation is considered the gold standard method for *Braschyspira* spp. diagnosis [22]. In the present study, isolation on selective agar also had the highest diagnostic sensitivity, detecting 14/16 and 7/15 positive samples in CLIN and SUBCL groups, respectively. This finding corroborates the literature [2] demonstrating that isolation is the best method to diagnose spirochetes when compared to clinical signs, qPCR, gross lesions, histology, and FISH. At necropsy, scraping of the colon was the method with highest isolation index, 15 compared to 7 from the cecum (data not shown). The subclinical isolated used in this study demonstrated a reduced ability (at least 50%) to infect and colonize susceptible pigs when compared to a clinical isolate. It is possible that the number of virulence factors harbored in these subclinical strains might be present in lower number. This hypothesis might be possible to be demonstrated in silico evaluation of the whole genome sequence comparing to clinical strains.

The pathogenesis of SD is complex and not fully elucidated, mainly because the disease is multifactorial. The infection depends on others anaerobic bacteria species in the large intestine, which contribute to colonization, induce extensive inflammation and necrosis of the epithelial surface of the cecum and colon [2,23]. Several virulence factors have been described for *B. hyodysenteriae*, including chemotaxis, motility, adhesion, hemolysin production and lipooligosaccharide (LOS) endotoxic activity [24,25]. 

*B. hyodysenteriae* hemolysis is considered one of the main virulence factors [26] and this phenotypic laboratory characteristic is often used to determine the clinical potential of isolates from SD clinical cases [27]. Based on complete genome analysis of *B. hyodysenteriae,* seven potential hemolysin genes were described [28]. The possible involvement of beta hemolysis in the pathogenesis of the disease has been evaluated in several studies [29,30,31]. However, its evaluation in clinical trials associated with the onset of intestinal lesions is scarce, and its importance in vivo is not fully elucidated. Thomson et al. [8] and Lysons et al. [7] compared virulent and potentially low virulence *B. hyodysenteriae* strains and reported difference in blood agar growth with poor hemolysis associated to potentially low virulence isolates. In other studies, in vitro hemolytic capacity was evaluated and described differences in hemolytic intensity in different strains of *B. hyodysenteriae* recovered form cases of SD [32,33]. However, analyzing isolates recovered from apparently healthy farms with no clinical signs of SD, no genetic and phenotypic differences in hemolysis were observed when compared to those isolated from clinically affected animals [11]. In the present study, both the CLIN and SUBCL isolates had the same phenotypic characteristics of strong hemolysis in blood agar and the same ST 245 using the MLST analysis [13]. This study does demonstrate that hemolysis on blood agar and sequence typing are not sufficient to determine the virulence of different typical strongly beta-hemolytic clinical or subclinical isolates of *B. hyodysenteriae*.

The subclinical isolate used in the present study was obtained from a farm of high healthy status and with no use of antimicrobials that could mask signs of the disease. The in vivo inoculation in pigs of this isolate demonstrated differences in the number of affected animals and beginning of clinical signs, however, it is important to consider the fact that this strain can colonize, induce lesions and clinical signs characteristic of SD in some animals. These findings raise great concern related to the necessity to screen replaced animals while in the quarantine, and the real important of having a quarantine, not only justified by PRRSv, PEDv, TGEv or *Mycoplasma hyopneumoniae*, but also for *B. hyodysenteriae.*

Although reported, it is unknown how long subclinical infection may persist in a herd, leading to the assumption that other apparently healthy herds may be similarly infected but remain undiagnosed. This is an important fact, especially when it may happen in nucleus or multiplier breeding herds of high healthy status, as they can carry the spirochete to other farms and spread the disease in conditions where there are healthy and management challenges [11]. Isolates recovered from pigs in apparently healthy multiplier herds has been described in Germany, Swiss and Australia [11,20,34,35], highlighting the epidemiological risk of these herds.

Serological tests were performed to identify herds with SD and could be an option to diagnosed subclinical infected herds, but there are no commercial kits that can be used. Based on LOS, *B. hyodysenteriae* has 11 serotypes widely distributed in different geographic regions. Genetic variation and marked differences in antigenic proteins are significant limitations for development of a globally applicable serological test [36]. Some studies using recombinant proteins have been carried out to develop a serological test that would be an important tool for detecting *B. hyodysenteriae* positive herds without clinical signs of SD [21,37].

The hypotheses to explain the presence of the SUBCL isolate in an apparently healthy animal from a herd with no clinical cases of SD but that developed clinical disease in an experimentally inoculated pig in the present study could be the following: (1) The SUBCL isolate had reduced virulence factors compared to typical clinical *B. hyodysenteriae* isolates, reducing the capability of proliferation and not reaching the infection level enough to cause disease and/or to be detected, and/or lacking virulence factors able to induce typical lesions [11,38,39]. (2) The source herd has a high healthy status with fewer challenges than a commercial herd, a condition that may influence spirochete colonization and disease development, or (3) The pressure of infection of *B. hyodysenteriae* among the host population on the farm was low and the amount of spirochetes used in the inoculum was sufficient to induce clinical disease in experimentally infected pigs [17]. Other aspects to be considered are the influence of feed ingredients and some substrates that might influence the microbiota and/or the physicochemical environment in the colon, which in turn may influence the ability of *B. hyodysenteriae* to colonize [39,40]. In addition, both isolates the current study, clinical and subclinical, were classified with the same ST 245; however, they were obtained from different farms, located in different regions (São Paulo and Minas Gerais States), so other unknown factors may be involved the clinical manifestation of SD.

## 5. Conclusions

This study clearly demonstrated that subclinical *B. hyodysenteriae* obtained from healthy animals in SD free herds may induce the disease, even with lower severity, when susceptible animals are exposed to high concentrations of the bacteria. Hemolysis or ST characteristics seems not to be definitive markers for pathogenicity and future in silico in association to in vivo studies are required to compared different subclinical and clinical isolates and evaluate possible determinants to disease development. Meanwhile, it is important to be aware about the existence of this subclinical *B. hyodysenteriae* and have strategies to minimize the chances of contamination of negative herds.

## Figures and Tables

**Table 1:** Fecal score, qPCR, bacterial isolation, gross lesions, microscopic lesion score and fluorescence in situ hybridization (FISH) of experimentally infected pig with clinical (CLIN), subclinical (SUBCL) of *Brachyspira hyodysenteriae* isolates and negative control (CTRL).

Group	Animal	5 DPI		7 DPI		11 DPI		15 DPI		18 DPI		*B. hyodysenteriae* Isolation	Gross Lesion	Microscopic Score	FISH *
		FE	qPCR	FE	qPCR	FE	qPCR	FE	qPCR	FE	qPCR				
CLIN	1	1	Neg	2	1.5 × 10^3^	4	2.9 × 10^7^	4	2.7 × 10^7^	4	1.4 × 10^7^	9 DPI	Yes	13	+
	2	2	8.5 × 10^3^	3.5	2.4 × 10^5^	4	1.2 × 10^8^	¥	¥	¥	¥	5 DPI	Yes	11.5	++
	3	1	Neg	2	Neg	2	Neg	2	Neg	1	Neg	18 DPI	No	3.5	-
	4	2	Neg	2	Neg	2	Neg	2	Neg	2	Neg	11 DPI	No	4.5	-
	5	2	Neg	1	Neg	2	Neg	2	Neg	2	Neg	11 DPI	No	3	-
	6	0	Neg	1	9.6 × 10^3^	4	3.4 × 10^7^	¥	¥	¥	¥	9 DPI	Yes	11	++
	7	1	Neg	2	Neg	2	3.5 × 10^3^	4	4.3 × 10^7^	3.5	2.6 × 10^7^	15DPI	Yes	10	++
	8	1	Neg	2	Neg	3	Neg	3	1.4 × 10^2^	3	7.4 × 10^4^	18 DPI	Yes	9.5	++
	9	1	Neg	2	Neg	4	4.4 × 10^7^	4	2.6 × 10^6^	4	2.5 × 10^7^	11 DPI	Yes	12.5	+++
	10	1	Neg	2	Neg	2	Neg	3	Neg	3	Neg	18 DPI	No	7	-
	11	1	Neg	3	Neg	3	Neg	3	Neg	3	Neg	11 DPI	No	4	-
	12	1	Neg	3	Neg	4	6.8 × 10^7^	4	7.6 × 10^7^	4	5.9 × 10^8^	11 DPI	Yes	14	++
	13	0	Neg	1	Neg	1	Neg	4	2.1 × 10^7^	¥	¥	15 DPI	Yes	14.5	++
	14	0	Neg	1	Neg	1	1.5 × 10^3^	0	2.5 × 10^3^	2	1.0 × 10^4^	Neg	No	1	-
	15	0	Neg	1	Neg	1	Neg	1	Neg	1	1.6 × 10^5^	Neg	No	1	-
	16	1	Neg	2	Neg	1	Neg	2	1.1 × 10^3^	2	7.2 × 10^7^	18 DPI	Yes	13.5	+++
SUBCL	17	0	Neg	0	Neg	1	Neg	0	Neg	0	Neg	Neg	No	0	-
	18	0	Neg	0	Neg	1	Neg	1	Neg	1	Neg	Neg	No	2.5	-
	19	0	Neg	2	Neg	1	Neg	2	Neg	1	Neg	Neg	No	7	-
	20	0	Neg	2	Neg	1	Neg	2	7.4 × 10^3^	2	1.5 × 10^3^	11 DPI	No	4	-
	21	0	Neg	0	Neg	2	Neg	0	Neg	0	Neg	Neg	No	2.5	-
	22	0	Neg	0	Neg	2	Neg	2	Neg	1	Neg	18 DPI	No	3	-
	23	0	Neg	0	Neg	2	Neg	2	Neg	1	Neg	Neg	No	6	-
	24	0	Neg	0	Neg	1	Neg	2	1.6 × 10^7^	2	3.0 × 10^4^	11 DPI	Yes	5	+++
	25	0	Neg	0	Neg	1	Neg	4	1.3 × 10^7^	4	2.5 × 10^7^	15 DPI	Yes	13.5	+++
	26	0	Neg	0	Neg	1	Neg	0	1.1 × 10^4^	2	2.1 × 10^5^	15 DPI	Yes	6.5	+
	27	0	Neg	2	Neg	1	Neg	2	Neg	1	Neg	Neg	No	0	-
	28	0	Neg	0	Neg	1	Neg	1	Neg	1	Neg	18 DPI	No	5	-
	29	0	Neg	0	Neg	1	Neg	1	Neg	1	Neg	18 DPI	No	1	-
	30	0	Neg	0	Neg	0	Neg	2	Neg	1	Neg	Neg	No	3	-
	31	1	Neg	0	Neg	2	Neg	2	Neg	2	Neg	Neg	Yes	2.5	-
CTRL	32	0	Neg	0	Neg	0	Neg	0	Neg	0	Neg	Neg	No	0	-
	33	0	Neg	0	Neg	0	Neg	0	Neg	0	Neg	Neg	No	0	-
	34	0	Neg	0	Neg	1	Neg	0	Neg	1	Neg	Neg	No	0	-
	35	0	Neg	0	Neg	0	Neg	0	Neg	0	Neg	Neg	No	0	-
	36	0	Neg	1	Neg	0	Neg	0	Neg	0	Neg	Neg	No	0	-
	37	0	Neg	0	Neg	0	Neg	0	Neg	0	Neg	Neg	No	0	-
	38	0	Neg	0	Neg	0	Neg	0	Neg	0	Neg	Neg	No	0	-
	39	0	Neg	0	Neg	1	Neg	1	Neg	1	Neg	Neg	No	0	-
	40	0	Neg	0	Neg	0	Neg	1	Neg	1	Neg	Neg	No	0	-
	41	0	Neg	0	Neg	1	Neg	1	Neg	1	Neg	Neg	No	0	-
	42	0	Neg	0	Neg	0	Neg	0	Neg	0	Neg	Neg	No	1	-
	43	0	Neg	0	Neg	0	Neg	0	Neg	0	Neg	Neg	No	0	-
	44	0	Neg	1	Neg	1	Neg	0	Neg	1	Neg	Neg	No	0	-
	45	0	Neg	0	Neg	0	Neg	0	Neg	0	Neg	Neg	No	1	-
	46	1	Neg	1	Neg	1	Neg	1	Neg	1	Neg	Neg	No	0	-
	47	0	Neg	0	Neg	0	Neg	0	Neg	0	Neg	Neg	No	0	-

DPI: days post inoculation; FE: fecal score; FISH: fluorescence in situ hybridization; * [-] No fluorescent labelling of *B. hyodysenteriae*, [+] mild fluorescent labeling of *B. hyodysenteriae*, [++] moderate fluorescent labeling of *B. hyodysenteriae*, [+++] severe fluorescent labeling of *B. hyodysenteriae*; ¥: euthanized animals.

**Table 2:** Clinical signs, macroscopic and microscopic lesions of swine dysentery after inoculation of *Brachyspira hyodysenteriae* in swine model.

	Clinical Signs *			Gross Lesions *			Histologic Lesions
Group	Aqueous and/or Mucohemorrhagic Diarrhea	First Observation of Diarrhea	Total Number of Days with Diarrhea	Excessive Mucus in the Lumen	Mucosal Hemorrhage	Fibrinous Exudate	Mean of the Final Score ± SD
Negative	0/16 ^a^	- **	0 ^a^	0/16 ^a^	0/16 ^a^	0/16 ^a^	0.02 ± 0.04 ^a^
Subclinical	1/15 ^a^	15 DPI	3 ^b^	3/15 ^a^	1/15 ^a^	1/15 ^a^	0.81 ± 0.39 ^b^
Clinical	10/16 ^b^	7 DPI	11 ^b^	9/16 ^b^	9/16 ^b^	8/16 ^b^	1.66 ± 0.26 ^c^

* Number of animals affected/total of animals; ** No diarrhea was observed among negative control pigs; ^a,b,c^: different letters indicate statistical differences between groups; DPI: days post inoculation; SD: standard deviation; Kruskal–Wallis test followed by Mann–Whitney test if significant.

## Data Availability

The data presented in this study are available by reasonable request from the corresponding author.
